# Two‐Factor Authentication Inspired Optical Security of Information Enabled by Silica Nanofibers

**DOI:** 10.1002/smll.202502697

**Published:** 2025-04-21

**Authors:** Zhen Lin, Xiaoqi Cui, Xiaodan Hong, Olli Ikkala, Bo Peng

**Affiliations:** ^1^ Department of Applied Physics Aalto University P.O. Box 15100 Espoo 02150 Finland; ^2^ Department of Materials Science Advanced Coatings Research Center of Ministry of Education of China Fudan University Shanghai 200433 China; ^3^ QTF Centre of Excellence Department of Electronics and Nanoengineering Aalto University Espoo 02150 Finland

**Keywords:** encryption−decryption, fluorescence, liquid‐ and light‐responsive, silica nanofiber, two‐factor authentication

## Abstract

Two‐factor authentication (2FA) is widely used in informatics for identity verification and information encryption, yet its applications in materials science remain largely underexplored. In this study, a composite film composed of silica nanofibers (NFs) and fluorescent nanofibers (FNFs) is presented, offering a unique material‐based approach to 2FA encryption. NFs and FNFs are synthesized via water‐in‐oil emulsions, resulting in films that can be both liquid‐ and light‐responsive. NFs assembly exhibits intriguing light‐scattering characteristics, rendering it opaque under normal conditions but transparent when wetted, functioning as a liquid‐triggered optically shielding material. Embedded FNFs remain concealed within NF matrix, becoming fluorescently visible only under ultraviolet A (UVA) light illumination of specific wavelength. The encryption system requires two decryption keys − liquid and UVA light, which must be applied sequentially to successfully access the encoded information. Upon liquid exposure, the film transitions from opaque to transparent, allowing light transmission. Subsequent UVA irradiation reveals the hidden fluorescent patterns labeled by FNFs in film. This sequential encryption−decryption mechanism mimics the principles of 2FA in digital data systems, providing a promising new paradigm for secure information storage and transmission for patterns using material‐based strategies.

## Graphical abstract

Inspired by the principles of two‐factor authentication in data informatics, a material‐based optical security encryption−decryption system is developed using silica nanofibers and fluorescent nanofibers. The engineered film exhibits unique liquid‐ and light‐responsiveness, enabling two‐level sequential encryption and decryption, which provides a promising new paradigm for secure information storage and transmission.

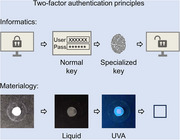

## 1 Introduction

**Figure 1:**
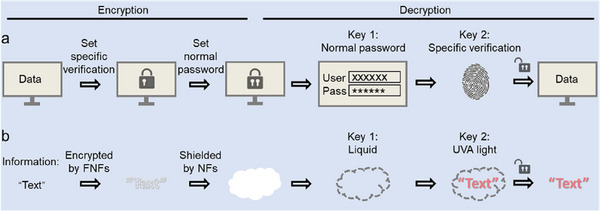
Schematic illustration of a) 2FA principles in digital data informatics and b) material‐based 2FA pattern encryption−decryption system using silica FNFs and NFs.

In the era of information explosion, data confidentiality has become increasingly important, especially for sensitive contents. Considerable efforts have been devoted to develope efficient strategies for identity verification and information encryption by intricate data algorithms, among which two‐factor authentication (2FA) gains particular attention. It requires two different types of credentials to confirm identity or access resources,^[^ ^1^, ^2^, ^3^ ^]^ such as, a password and biometric data.^[^ ^4^, ^5^ ^]^ Unlike single encryption, 2FA incorporates an additional specialized layer of protection, ensuring that even if passwords are compromised, further credentials are needed for access. This approach employs sequential encryption: a general outer layer (the “what you know” factor) and a specialized inner layer (the “what you have” or “what you are” factor) (**Figure** **1**a).

Drawing an analogy to information encryption while in material field, single encryption relies on a material's singular responsive properties to secure information, with a single trigger required for decryption. The approach can allow efficient pattern encryption−decryption. Therein, fluorescent materials encode data that remains concealed until exposed to light at a matched excitation wavelength, causing fluorescence to reveal the information.^[^ ^6^, ^7^ ^]^ While simple and convenient, single encryption offers low security.

To address this limitation, advanced encryption strategies have emerged, including 3D coded encryption,^[^ ^8^, ^9^ ^]^ multi‐color anti‐counterfeiting,^[^ ^10^, ^11^ ^]^ multi‐component encryption,^[^ ^12^ ^]^ and dynamic encryption.^[^ ^13^ ^]^ These approaches involve spatiotemporal complexity for decryption, significantly enhancing security.^[^ ^14^, ^15^, ^16^ ^]^ Such strategies often employ multi‐responsive materials – typically optical materials paired with other stimuli‐responsive entities, capable of reacting to diverse external stimuli like pH,^[^ ^17^ ^]^ temperature,^[^ ^18^ ^]^ magnetism,^[^ ^19^ ^]^ or mechanical force,^[^ ^20^ ^]^ either individually or simultaneously.^[^ ^21^, ^22^ ^]^ Despite their advancements, these methods usually entail intricate material synthesis and/or strict selection of stimuli.

In contrast, while 2FA‐based encryption may not offer the absolute highest level of security compared to those advanced encryption methods, it is far more practical and widely used in everyday life.^[^ ^1^, ^2^, ^3^, ^4^, ^5^ ^]^ For example, 2FA is commonly used in email and account logins because it offers a balance between security and usability. Its logic is straightforward and easy to understand, while its encoding remains simple and computationally efficient. More importantly, 2FA can provide a sufficient level of security for most applications without requiring overly complex encryption algorithms, specialized hardware, or intricate decryption processes. This practicality makes 2FA as a preferred choice in real‐world security applications, where efficiency and accessibility are just as important as protection. Similarly, in material field, 2FA‐based encryption relies on simpler materials and processes than those advanced encryption methods, reducing cost, complexity, labor, time, and environmental impact. Meanwhile, maintaining a certain level of security, such as enforcing the correct sequence of key usage, ensures the system remains both effective and reliable. By balancing security with practicality, 2FA‐based encryption can become a truly valuable solution for real‐world applications.

In this work, we showcase a straightforward pathway to allow 2FA‐based information encryption using a composite film constructed from silica nanofibers (NFs) and fluorescent nanofibers (FNFs) (Figure 1b). Silica NFs and FNFs are selected for their accessibility, environmental friendliness, and facile synthesis, offering a simpler and more sustainable alternative to conventional opaque and fluorescent materials. The encrypted information resides within FNFs, which are hidden by a white NF layer, rendering the composite film opaque and concealing the information.^[^ ^23^ ^]^ Decryption requires two sequential keys: liquid first, making the film transparent, followed by ultraviolet A (UVA) light at ≈400 nm, which reveals the fluorescence. The liquid‐responsive layer acts as a universal, low‐security key, while UVA light serves as a highly secure, specialized key. Any liquid with a refractive index closing/matching that of NFs can serve as the first key.^[^ ^24^ ^]^ This liquid responsiveness is primarily driven by changes in refractive index contrast rather than specific chemical compositions or structural transformations, which is highly versatile and widely applicable. However, only UVA light at a specific wavelength can act as the second key, exciting the fluorescence for final decryption. This material‐based encryption closely mimics 2FA principles in digital security, enforcing stepwise decryption unlike conventional systems with independent steps. Additionally, the silica‐based platform is highly accessible, cost‐effective, and scalable, offering a practical balance between security and usability for applications.

## 2 Results and Discussion

### 2.1 Synthesis and Characterization of Silica NFs and FNFs

The silica NFs are synthesized via the water‐in‐oil emulsion, wherein randomly curly morphology is driven by non‐synchronized Brownian‐motions between template droplets and silica NFs.^[^ ^23^ ^]^ Briefly, silica sprouts from small aqueous droplets stabilized by sodium citrate in pentanol, where the hydrolysis and condensation of tetraethyl orthosilicate (TEOS) precursors drive growth (Figure S1a, Supporting Information). Both droplets and silica undergo non‐synchronized Brownian motions because of size discrepancy between droplets and silica. Consequently, curly NFs with irregular morphologies are formed after the silica precursors are depleted.

**Figure 2:**
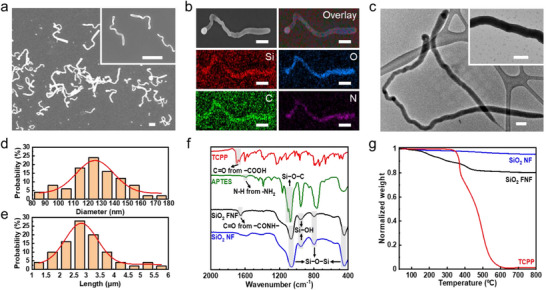
Characterization of silica FNFs. a) Scanning electron microscopy (SEM) images of FNFs. Both scale bars are 2 µm. b) Elemental mapping analysis of an FNF. All scale bars are 500 nm. c) Transmission electron microscopy (TEM) observation of FNFs. Both scale bars are 200 nm. d) Diameter and e) length distribution histograms of FNFs with normal fitting (red curves). f) ATR‐FTIR spectra of TCPP, APTES, NFs, and FNFs. g) TGA of TCPP, NFs, and FNFs.

Similarly, curly silica FNFs are prepared when fluorescently modified silica precursors are incorporated alongside TEOS. In this work, tetrakis(4‐carboxyphenyl) porphyrin (TCPP) is grafted onto 3‐aminopropyl triethoxysilane (APTES) to synthesize the fluorescent precursor (APTES‐TCPP), which is then used for FNF fabrication (Figure S1b, Supporting Information). Like pristine silica NFs (Figure S2, Supporting Information), FNFs remain randomly curly morphology (**Figure** **2**a; Figure S3, Supporting Information). Elemental mapping shows significant enrichment of C and N in FNFs (Figure 2b), confirming the successful incorporation of APTES and TCPP.^[^ ^25^ ^]^ Additionally, FNFs are internally solid (Figure 2c) and amorphous (Figure S4, Supporting Information). Two structural parameters, i.e., the diameter and length, are measured and presented as histograms in Figure 2d,e, respectively. The average diameter and length are 127.5 nm and 2.93 µm, respectively. These worm‐like 1D structures, characterized by their large aspect ratio, facilitate the random weaving of FNFs into stable, porous networks within composite films.

The chemical structures of FNFs are analyzed using attenuated total reflectance‐Fourier transform infrared (ATR‐FTIR) spectra, as shown in Figure 2f. NFs exhibit four characteristic peaks at 1060, 950, 800, and 460 cm^−1^, attributed to the antisymmetric stretching vibration of Si−O−Si, vibration of Si−OH, symmetric stretching vibration of Si−O−Si, and bending of Si−O−Si, respectively.^[^ ^26^, ^27^ ^]^ In addition to these peaks, FNFs show a distinctive peak at 1650 cm^−1^, belonging to the vibration of C═O from −CONH− groups.^[^ ^26^, ^27^ ^]^ This peak is slightly shifted from the C═O vibration of −COOH groups in TCPP molecules, which typically appears at 1700 cm^−1^. Simultaneously, the deformation vibration peak of N−H from −NH_2_ groups at 1590 cm^−1^ diminishes in the spectrum of FNFs, indicating complete amidation between −COOH and −NH_2_ groups. This confirms that TCPP molecules are chemically grafted onto FNFs through covalent bonding, rather than merely blending.^[^ ^26^, ^27^ ^]^ The stable chemical bonds in FNFs enhance their structural robustness and long‐lasting fluorescence stability, especially in TCPP‐soluble organic solvents, significantly broadening the potential applications of FNFs in liquid environments or under wet conditions.

The TCPP content in FNFs is estimated based on the weight loss determined by thermogravimetric analysis (TGA).^[^ ^27^ ^]^ As an organic compound, TCPP begins to decompose at ≈300 °C and is almost entirely degraded by 600 °C (red line, Figure 2g). In contrast, silica NFs show only minimal weight loss (4.5 wt.%) in this temperature range, primarily due to the evaporation of bound water (blue line, Figure 2g). For silica FNFs, a total weight loss of 19.7 wt.% is observed upon heating to 800 °C, which includes both the decomposition of TCPP and a small contribution from bound water (black line, Figure 2g). By subtracting the weight loss attributed to bound water, the remaining weight loss in FNFs corresponds to the grafted organic phase, predominantly TCPP. This analysis indicates that the TCPP content in FNFs is ≈15 wt.%.

### 2.2 Fluorescent Properties of Silica NFs and FNFs

**Figure 3:**
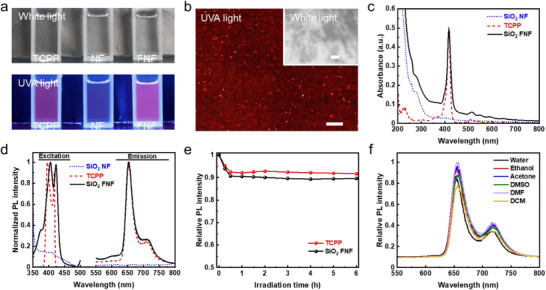
Fluorescent properties of silica FNFs. a) Photographs of TCPP solution, NF dispersion, and FNF dispersion in ethanol under white and UVA lights. b) Confocal optical images of FNFs under white light and UVA irradiation. Scale bars are 2 µm. c) Absorption spectra and d) PL spectra of TCPP solution, NF dispersion, and FNF dispersion in ethanol. e) Relative PL intensity decay of TCPP solution and FNF dispersion under continuous UVA light illumination. f) PL emission spectra of FNF dispersion in different solvents. The concentration of FNFs remains 1 mg mL^−1^.

Photographs in **Figure** **3**a show the appearance of TCPP solution, NF dispersion, and FNF dispersion in ethanol under white light and UVA light, using a commercial dual‐use 6‐Watt illuminator (Figure S5, Supporting Information). All samples appear transparent and colorless under white light, inferring that visible light does not induce fluorescence. Under UVA light, however, TCPP solution and FNF dispersion emit a distinct red fluorescence, while the non‐fluorescent NF dispersion remains colorless.

In Figure 3b, microscopic FNFs exhibit red fluorescence when excited by UVA light, while under visible light, they appear white. Interestingly, the red fluorescence intensity is noticeably higher at one end of FNFs, suggesting a longitudinal gradient of TCPP concentration. This phenomenon can be attributed to the growth mechanism of FNFs. During synthesis, silica solidifies preferentially at one side of the emulsion droplets, where the concentration of APTES‐TCPP is initially higher.^[^ ^23^, ^28^, ^29^ ^]^ As FNFs grow, APTES‐TCPP is gradually consumed, resulting in a decreasing concentration gradient along FNF length, and consequently, a reduction in fluorescent intensity from head to tail.

The fluorescent properties of TCPP, NFs, and FNFs in ethanol are next studied. NFs without TCPP are non‐fluorescent and does not absorb visible light or UVA light (Figure 3c). In contrast, TCPP and FNFs exhibit fluorescence, primarily absorbing light at ≈420 nm, with minor absorptions at ≈510, ≈550, ≈590, and ≈640 nm (Figure 3c), consistent with previous reports.^[^ ^30^, ^31^, ^32^ ^]^ FNFs share the similar excitation (≈405 and ≈420 nm) and emission (≈650 and ≈715 nm) wavelengths as TCPP (Figure 3d). However, a slight redshift in the excitation peaks of FNFs is observed, likely due to the encapsulation of TCPP within the silica matrix.^[^ ^27^ ^]^ The fluorescence of FNFs is remarkably stable, with only 10% photoluminescence (PL) intensity decay under continuous UVA exposure for 6 h (Figure 3e; Figure S6, Supporting Information). Moreover, the fluorescence persists in various solvents, demonstrating the versatility of FNFs (Figure 3f). In summary, while NFs are non‐fluorescent, FNFs effectively absorb UVA light and emit stable, long‐lasting red fluorescence across different solvents. These properties make FNFs highly promising for applications in fluorescent painting and secure information encryption.

### 2.3 Optical Responsiveness of Silica NF, FNF, and NF/FNF Films

The refractive index plays a crucial role in determining the scattering properties of materials. For biphasic heterogeneous materials, the contrast in refractive indexes between two phases significantly affects the bulk scattering performance, such as opacity.^[^ ^33^, ^34^, ^35^ ^]^ Decreasing this contrast can transform opaque materials into transparent ones. For example, porous opaque materials often become transparent when wetted by liquids, as liquids typically have refractive indexes closer to those of solids in contrast to air. This reduces light scattering and enhances light transmission. Herein, the opacity is evaluated by measuring the intensity of UV–vis reflected light and transmitted light, quantified as reflectance and transmittance (Figure S7, Supporting Information). These measurements are performed using an integrating sphere (Figure S8, Supporting Information), with water used as the wetting agent for the films.

**Figure 4:**
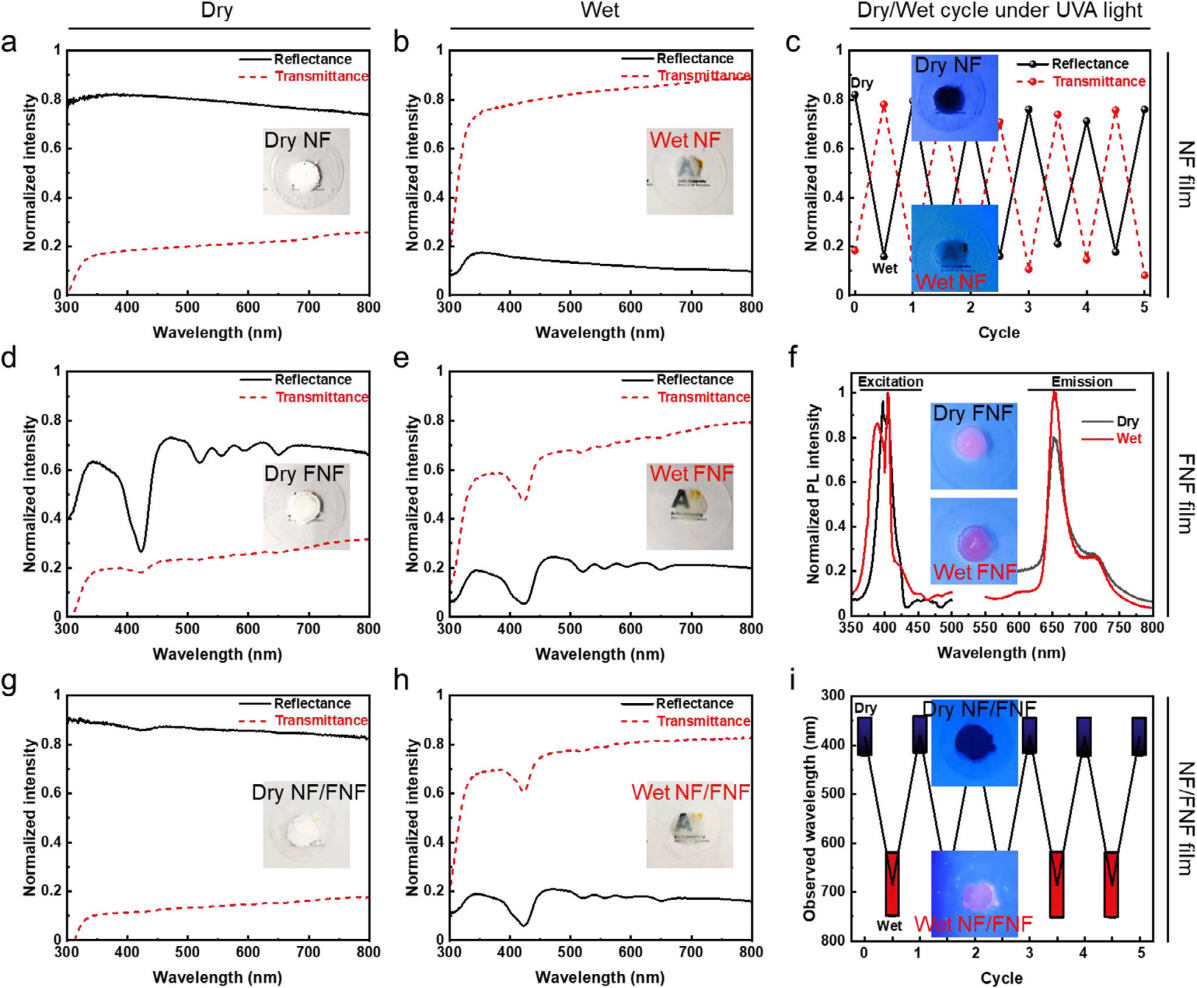
Optical responsiveness of silica NF, FNF, and NF/FNF films at dry and wet states. a,b) Reflectance and transmittance of NF film under dry and wet condition. c) Reflectance and transmittance changes of NF film under UVA light in alternating dry/wet cycles. d,e) Reflectance and transmittance of FNF film under dry and wet condition. f) PL spectra of dry/wet FNF film under UVA light. g,h) Reflectance and transmittance of NF/FNF film under dry and wet condition. i) Color changes of NF/FNF film under UVA light in alternating dry/wet cycles.

Silica nanoparticles typically have a significantly higher refractive index (≈1.445)^[^ ^28^ ^]^ compared to air (≈1.000), giving NF film high reflectance and low transmittance for visible light (**Figure** **4**a). However, when NF film is wet with water, it becomes much more transparent, as its reflectance drops sharply while transmittance increases dramatically (Figure 4b). This occurs because water penetrates the pores of the film, replacing air and decreasing the refractive index contrast within the film. The refractive index of water (≈1.333) is much closer to that of silica, significantly weakening the film's light‐scattering ability.

This effect is not limited to water; other common organic solvents, e.g., acetone, ethanol, dichloromethane (DCM), *N*, *N*‐dimethylformamide (DMF), and dimethyl sulfoxide (DMSO), which have refractive indexes ≈1.4, produce a similar transparency effect (Figure S9, Supporting Information). This opacity−transparency change is reversible, as the film reverts to its opaque state upon drying. Under UVA light, this transition is similar (Figure 4c): dry NF film reflects UVA light and appears dark blue, while wet film becomes transparent. This reversible opacity shift can serve as a relatively simple, low‐security encryption method. Any liquid with a refractive index close to that of NFs can unlock the film's encryption. Consequently, the liquid‐responsive NF film is suitable as an outer lock for non‐information‐related shielding in the sequential encryption system, protecting the more secure inner lock. This liquid‐responsiveness corresponds to the concept of the general outer‐layer encryption in informatics 2FA, such as character‐based passwords, which lack specific selectivity or uniqueness but enhance practicality, representing the “what you know” factor.

Similarly, FNF film exhibits high reflectance and low transmittance when dry (Figure 4d) but becomes transparent when exposed to liquid (Figure 4e). The spectral patterns are attributed to light absorption by TCPP molecules at specific wavelengths. For instance, the most significant drop occurs at ≈420 nm, which corresponds to the primary absorption peak of TCPP and FNFs (consistent with Figure 3c). Unlike the dark blue appearance of NF film under UVA light, FNF film emits red fluorescence regardless of whether it is dry or wet (Figure 4f). This indicates that the fluorescence of FNFs remains stable even in the presence of liquid. Consequently, FNF film, with its robust fluorescent property, is well‐suited for use as an inner lock in an encryption system, enabling control over information concealment and revelation using white light and UVA light respectively. This light‐responsive encryption offers a significantly higher level of security compared to liquid‐responsive encryption. Decryption is only possible with precise knowledge of the specific light wavelength (e.g., UVA) and access to the appropriate illumination equipment. This light‐responsiveness parallels the concept of the specific inner‐layer encryption in informatics 2FA, like short messaging service verification codes or personal fingerprints, which are uniquely tied to an individual, representing the “what you have” or “what you are” factor.

Lastly, by assembling NFs on FNFs, NF/FNF composite film is constructed. When dry, the composite film exhibits spectra like those of NF film (Figure 4g), while under liquid exposure, its behavior resembles that of FNF film (Figure 4h). This transition from opaque to transparent upon wetting allows the inner FNFs to absorb specific wavelengths of light and emit fluorescence. Consequently, NF/FNF film undergoes a reversible color change between dark blue (Figure S10, Supporting Information) and red (Figure S11, Supporting Information) under UVA light during alternating dry/wet cycles (Figure 4i). These dual liquid‐ and light‐responsive properties makes the composite film particularly suitable for 2FA‐based optical encryption.

### 2.4 2FA‐based Optical Security of NF/FNF Film

**Figure 5:**
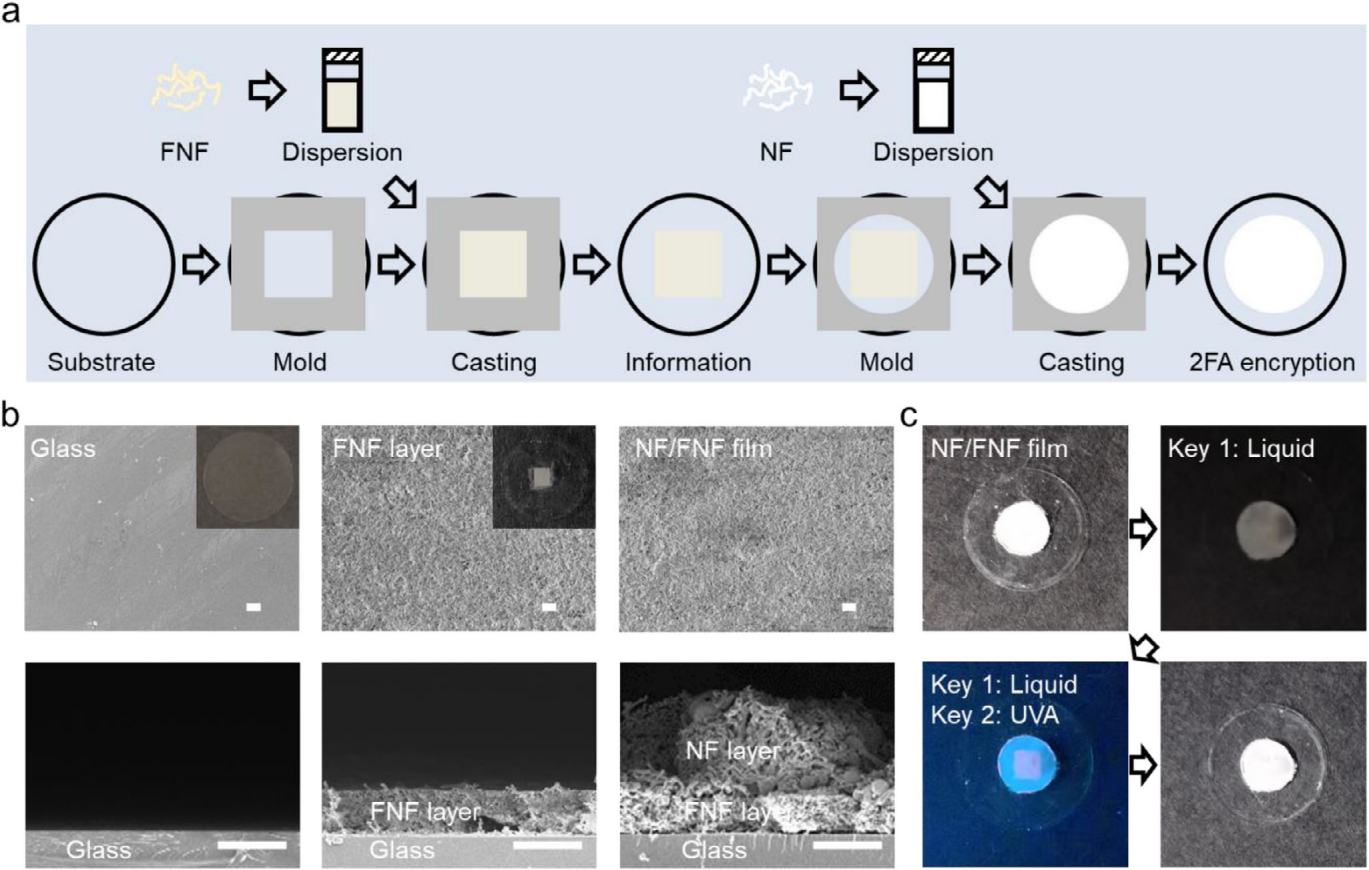
Encryption and decryption of information in the NF/FNF film with 2FA‐based optical security. a) Schematics of encrypting information in the NF/FNF film. b) Photographs and SEM images of encryption process. All scale bars are 10 µm. c) Information decryption using water and UVA light in sequence.

Information can be encrypted into the NF/FNF film using hollow molds to create specific patterns. For instance, **Figure** **5**a and Figure 5b demonstrate the creation of NF/FNF film with simple graphic information on a glass substrate. First, a square‐shaped hollow mold is placed on the glass substrate, and FNF dispersion is added. After the solvent evaporates and the mold is removed, a square‐shaped FNF layer is left behind, containing the graphic information of “square” (Figure 5b). Next, a rounded hollow mold (or another unrelated shape) is used to apply a layer of NF dispersion over the FNF layer. After drying, this process forms NF shielding layer on top of the FNF layer (Figure 5b). The final NF/FNF film has a white, rounded appearance (top left photo in Figure 5c), completely concealing the underlying the graphic information of “square”. This design ensures effective optical encryption by masking the encrypted information with the NF layer.

The encrypted information in the NF/FNF film is decrypted using 2FA keys sequentially: liquid and UVA light. Initially, when the film is dry and viewed under common light, no information or misleading information, such as the visible “round” shape – can be observed on the surface (top left photo in Figure 5c). Upon wetting the film with liquid, e.g., water, it turns transparent, but the true encrypted information remains hidden (top right photo in Figure 5c). When this wet film is then illuminated with UVA light, it emits red fluorescence, revealing the true encrypted information, such as the “square” pattern (bottom left photo in Figure 5c). Remarkably, removing these two decryption keys reverses the process. The NF/FNF film dries, returning to its opaque state and effectively concealing the information once again (bottom right photo in Figure 5c). Compared to the system with only the FNF layer where the pattern is both directly visible and easily readable under UVA light, the system with an additional NF shielding layer effectively conceals the pattern from direct observation. Moreover, it prevents the inner‐layer FNFs from being exposed to UVA excitation light, thereby safeguarding the true information (Figure S12, Supporting Information). This setup illustrates that our system follows the 2FA principle, requiring a sequential decryption process: first using liquid to unlock the outer layer, followed by light to decrypt the inner layer. This sequential process enhances security.

**Figure 6:**
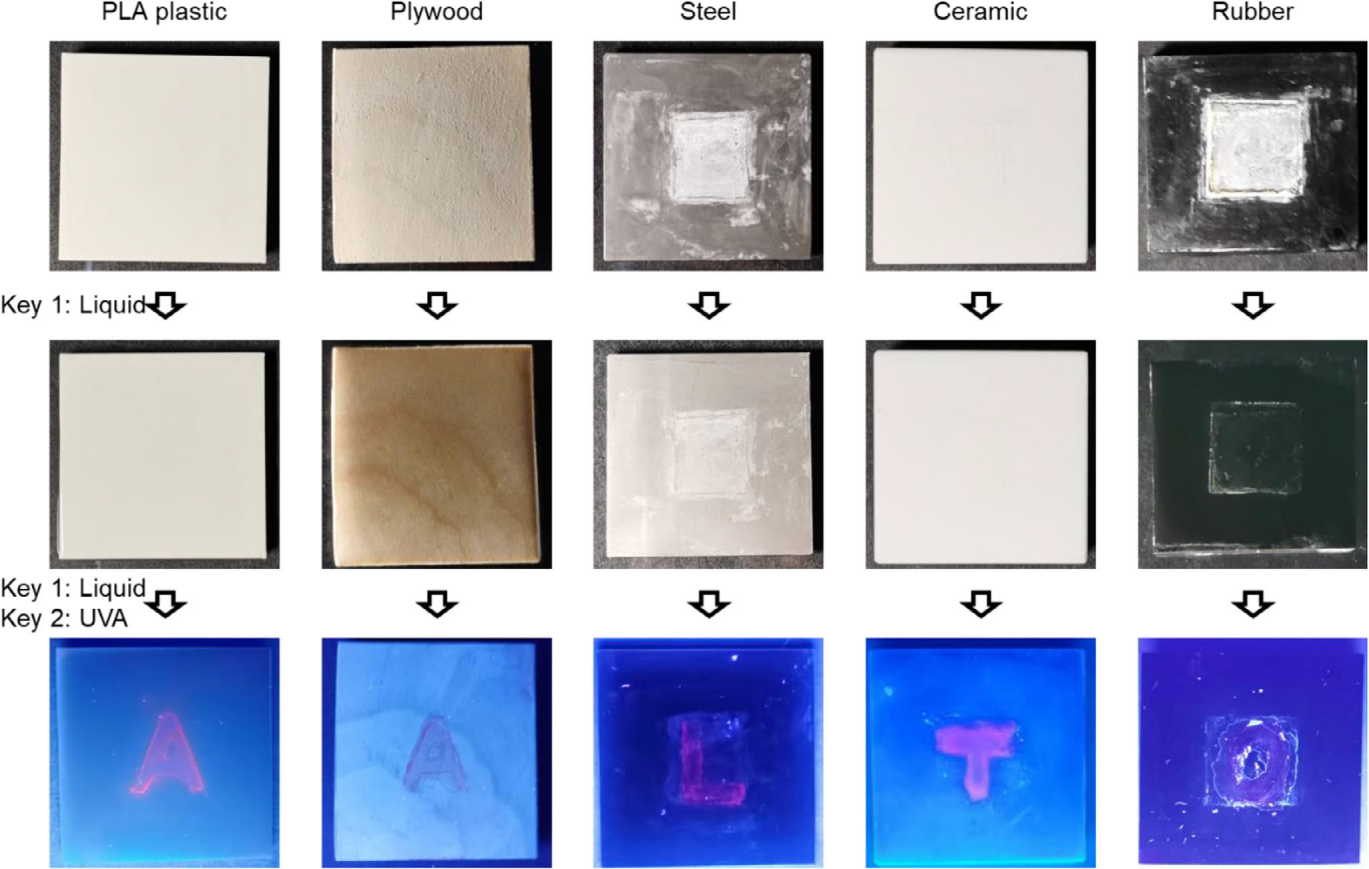
Alternative substrates and information formats. Substrates can be PLA plastic, plywood, steel, ceramic, rubber, etc. Information can be in textual format by patterns.

Finally, the application of this 2FA‐based encrypted optical security approach is further extended to textual‐format information and various substrates in daily life. Five substrate types (5 cm × 5 cm each) are demonstrated, including polylactic acid (PLA) plastic, biomass‐derived plywood, metal steel, inorganic ceramic, and black‐colored rubber (**Figure** **6**). Pattern information, in the form of alphabets, is encrypted using FNFs and concealed beneath the NF layer. When the first key, liquid, is applied, the films become transparent, but the concealed information remains hidden. However, when the second key, UVA light, is introduced, the encrypted alphabets are revealed, spelling out the textual information “AALTO”. Additionally, encrypting patterns on curved surfaces is also feasible when using soft molds. For example, an encryption−decryption logo can be fabricated on the curved surface of a daily‐use cup (Figure S13, Supporting Information). Moreover, the patterns prepared using this method exhibit long‐term stability. For example, the ceramic sheet in Figure 6, which secretes the “T” alphabet, still reveals the same information over two years of storage, despite some fluorescence fading (Figure S14, Supporting Information). Furthermore, owing to the stability of silica, this method exhibits strong resistance to harsh environmental conditions. Even after exposure to high humidity, elevated temperature, or subzero temperature, the encryption system remains stable (Figure S15, Supporting Information). The resolution limit of this approach is ≈3 mm (Figure S16, Supporting Information), which is sufficiently fine for unaided visual detection and suitable for various practical applications. For scenarios demanding higher resolution or finer patterning, alternative fabrication techniques, such as inkjet printing or photopatterning, may be required to achieve further enhancement. These demonstrations highlight the versatility and practicality of the 2FA‐based encryption−decryption optical security approach. It effectively encrypts and decrypts pattern and textual information across diverse substrates, making it suitable for real‐world applications.

There are many pioneering studies demonstrating a variety of encryption−decryption systems, such as single encryption, double encryption, multi‐color encryption, 3D encryption, dynamic encryption, and even 4D encryption (Table S1, Supporting Information). Simple encryption methods typically require accessible raw materials and straightforward fabrication steps but often exhibit low security levels, as they can be decrypted with a single stimulus. In contrast, advanced encryption techniques offer high security but rely on expensive materials (e.g., rare‐earth elements) and complex fabrication steps. The 2FA‐based encryption system in this work strikes a balance between security and practicality. It ensures a reliable security level while avoiding the complicated fabrication. Additionally, compared to hydrogel‐based systems that may require specific environmental conditions, and inkjet printing approaches that request special device, our system can be implemented via a simple coating process and adapted to various real‐world environments, enhancing its practicality. These advantages align with the principles of 2FA encryption, whilst emphasizing ease of use without compromising security. Therefore, our approach is particularly suitable for applications requiring moderate security with high encryption−decryption efficiency.

Overall, using the liquid‐ and light‐responsive properties of silica NFs and FNFs, this material‐based encryption−decryption system establishes a sequential security structure akin to 2FA principles in digital data informatics. The system is user‐friendly, versatile, highly repeatable, and requires minimal material resources, making it a promising solution for secure information storage and transmission in material field.

## 3 Conclusion

In summary, we present a flexible strategy for fabricating silica NF/FNF film with liquid‐ and light‐responsiveness, enabling two‐level sequential encryption−decryption inspired by 2FA in digital data informatics. Curly silica NFs and FNFs are synthesized via a water‐in‐oil sol‐gel method, and NF/FNF composite film is constructed by embedding FNFs within NF matrix. The surface NF layer is non‐fluorescent, scatters light, and appears opaque, effectively concealing the information. Upon exposed to liquids, it becomes transparent, allowing light transmission but offering a low level of security. In contrast, the embedded FNF layer is fluorescent, selectively absorbing UVA light and emitting red fluorescence, thereby controlling information concealment and revelation with a higher level of security. This 2FA‐inspired encryption−decryption method, realized through a simple, cost‐effective silica‐based design, ensures sequential security while maintaining ease of use and, thus is well‐suited for practical applications in everyday life.

## 4 Experimental Section

### Materials

TEOS (99.999%), APTES (99%), sodium citrate (sodium citrate tribasic dihydrate, 99%), *N*‐hydroxysuccinimide (NHS, 98%), *N*‐ethyl‐*N*’‐(3‐dimethylaminopropyl)carbodiimide hydrochloride (EDAC, 99%), and polyvinylpyrrolidone (PVP, average molecular weight Mn = 40000 g mol^−1^) were purchased from Sigma–Aldrich. 1‐pentanol (99%), ammonia (ammonium hydroxide, 25% in water), and DMF (99.8%) were obtained from *ACROS Organics*. Ethanol (99.5%) was purchased from *Altia Oyj*. TCPP (97%) was sourced from *Tokyo Chemical Industry*. All reagents were used as received. Ultrapure water (18.2 MΩ cm) was produced by a *Millipore direct‐Q* system, which was used in all experiments.

### Synthesis of Silica NFs

Silica NFs were synthesized using a water‐in‐oil sol‐gel method reported elsewhere.^[^ ^23^ ^]^ In detail, 1 g of PVP was dissolved in 10 mL of 1‐pentanol by sonication. Then, 0.25 mL water, 0.1 mL sodium citrate aqueous solution (0.18 mol L^−1^), 0.15 mL ammonia, and 2 mL ethanol were added into the solution, followed by violent shaking and sonication to form a well‐dispersed emulsion. Finally, 0.1 mL of TEOS was added dropwise into the emulsion and mixed by light shaking and sonication for 10 s. The mixtures were left to rest for reaction at room temperature for 6 h. After the reaction, the turbid mixtures were centrifuged at 1500 g for 30 min. The supernatant was removed while the sediment was redispersed in ethanol by sonication. This particle dispersion was centrifuged at 700 g for 15 min, followed by a new dispersive process with water. Again, it was centrifuged at the same condition. The whole centrifugation procedure was repeated at least 3 times to remove the reactant residue. Lastly, samples were dried naturally, and silica NF powder was yielded.

### Synthesis of Silica FNFs

Silica FNFs were synthesized via the similar method to silica NFs but incorporating APTES‐TCPP into TEOS. Specifically, 33.2 mg of APTES and 29.7 mg of TCPP were dissolved in 10 mL of DMF. 28.8 mg of EDAC and 17.3 mg of NHS were added to the solution, followed by stirring at room temperature for 24 h to get the dark‐red APTES‐TCPP solution which could be stored persistently away from light. When silica FNFs were synthesized, 0.1 mL of prepared APTES‐TCPP solution was added into water‐in‐oil emulsion accompanying with 0.1 mL of TEOS. The reaction conditions and separation processes remained. Finally, silica FNF powder was obtained, which exhibited significant fluorescent properties.

### Characterization of Silica NFs and FNFs

The morphologies of silica NFs and FNFs were observed using SEM (*Zeiss Sigma VP*), TEM (*JEOL JEM‐2200FS*), and atomic force microscopy (AFM, *Bruker Dimension Icon*) with a tapping mode. Image J was used to characterize the morphological parameters (e.g., diameter and length). The crystal structures and chemical structures were characterized by an X‐ray diffractometer (XRD, *Rigaku SmartLab*) and an ATR‐FTIR spectrometer (*Bruker Alpha II*), respectively. The TGA was performed on a TGA instrument (*TA Instrument Q500*) over a temperature range of 30 to 800 °C with a heating ramp of 10 °C min^−1^ in air.

### PL Experiments of Silica FNFs

The white and UVA lights were produced by a commercial dual‐use 6‐Watt illuminator (*Analytikjena UVL‐16*) with both mercury‐vapor white lamp and UVA lamp. Photographs of macroscopic solutions/dispersions and microscopic FNFs were taken in darkroom by camera and optical microscopy (*WITec Alpha300*) with confocal mode, respectively. UV–vis absorption spectra were recorded on a UV–vis spectrophotometer (*Agilent Cary 5000*). PL spectra were obtained from a fluorescence spectrometer (*Quanta Master 40*).

### Preparation of Silica NF, FNF, and NF/FNF Films

Sedimentation was used for preparing the films (Figure S17, Supporting Information). 5 mg of silica NF or FNF powder was dispersed in 1 mL of ethanol by sonication. A custom evaporation device was vertically assembled, consisting of three components: a flat base at the bottom, a glass sheet substrate (25 mm in diameter, *Menzel Glaser*) in the middle, and a funnel at the top. Ensuring the device leak‐free, the dispersion was carefully added into the funnel and allowed to evaporate ethanol completely. NF film or FNF film were obtained as NFs or FNFs were deposited onto glass substrates. The NF/FNF composite film was prepared similarly by sequentially depositing FNFs and NFs. These NF, FNF, and NF/FNF films were used for morphological and optical characterizations. Furthermore, to encrypt information into NF/FNF film, hollow molds with patterns were covered on glass substrates prior to the deposition process. The resulting information‐encrypted films were used for application demonstrations.

### Optical Properties of Silica NF, FNF, and NF/FNF Films

Photographs of films were taken by the camera under illumination with the illuminator. The surface and cross‐sectional structures of films were observed by SEM. For cross‐sectional observation, the samples were prepared by cryo‐fracturing films in liquid nitrogen. A 4 nm‐thick platinum layer was coated on the surface of samples before observation using a sputtering coater (*Leica EM ACE600*). The UV–vis reflectance and transmittance spectra of films were measured by the UV–vis spectrophotometer with an integrating sphere (*Agilent DRA 2500*). The PL spectra were obtained using the fluorescence spectrometer.

## Conflict of Interest

The authors declare no conflict of interest.

## Author Contributions

Z.L. and B.P. conceived the ideas. Z.L. synthesized the nanoparticles, fabricated the films, performed the characterizations, and made the analyses. X.C. and X.H. assisted to the experiments. O.I. and B.P. provided critical guidance on the experiments. Z.L., O.I., and B.P. wrote and revised the manuscript with support from the other co‐authors.

## Supporting information



Supporting Information

## Data Availability

The data that support the findings of this study are available from the corresponding author upon reasonable request.
