# Supplementary material for: Two‐Factor Authentication Inspired Optical Security of Information Enabled by Silica Nanofibers
Source: Small. 2025 Apr 21;21(23):2502697. doi: 10.1002/smll.202502697 (PMC12160666; doi:10.1002/smll.202502697)
Supplement: Supplementary file 1 — Supporting Information [file SMLL-21-2502697-s001.docx]

Supporting Information

Two-Factor Authentication Inspired Optical Security of Information Enabled by Silica Nanofibers

Zhen Lin, Xiaoqi Cui, Xiaodan Hong, Olli Ikkala, Bo Peng*

Zhen Lin, Xiaodan Hong, Olli Ikkala, Bo Peng

Department of Applied Physics, Aalto University, P.O. Box 15100, Espoo 02150, Finland.

E-mail: [pengbo006@gmail.com](mailto:pengbo006@gmail.com); peng_bo@fudan.edu.cn

Zhen Lin, Bo Peng

Department of Materials Science, Advanced Coatings Research Center of Ministry of Education of China, Fudan University, Shanghai 200433, China.

Xiaoqi Cui

QTF Centre of Excellence, Department of Electronics and Nanoengineering, Aalto University, Espoo 02150, Finland.


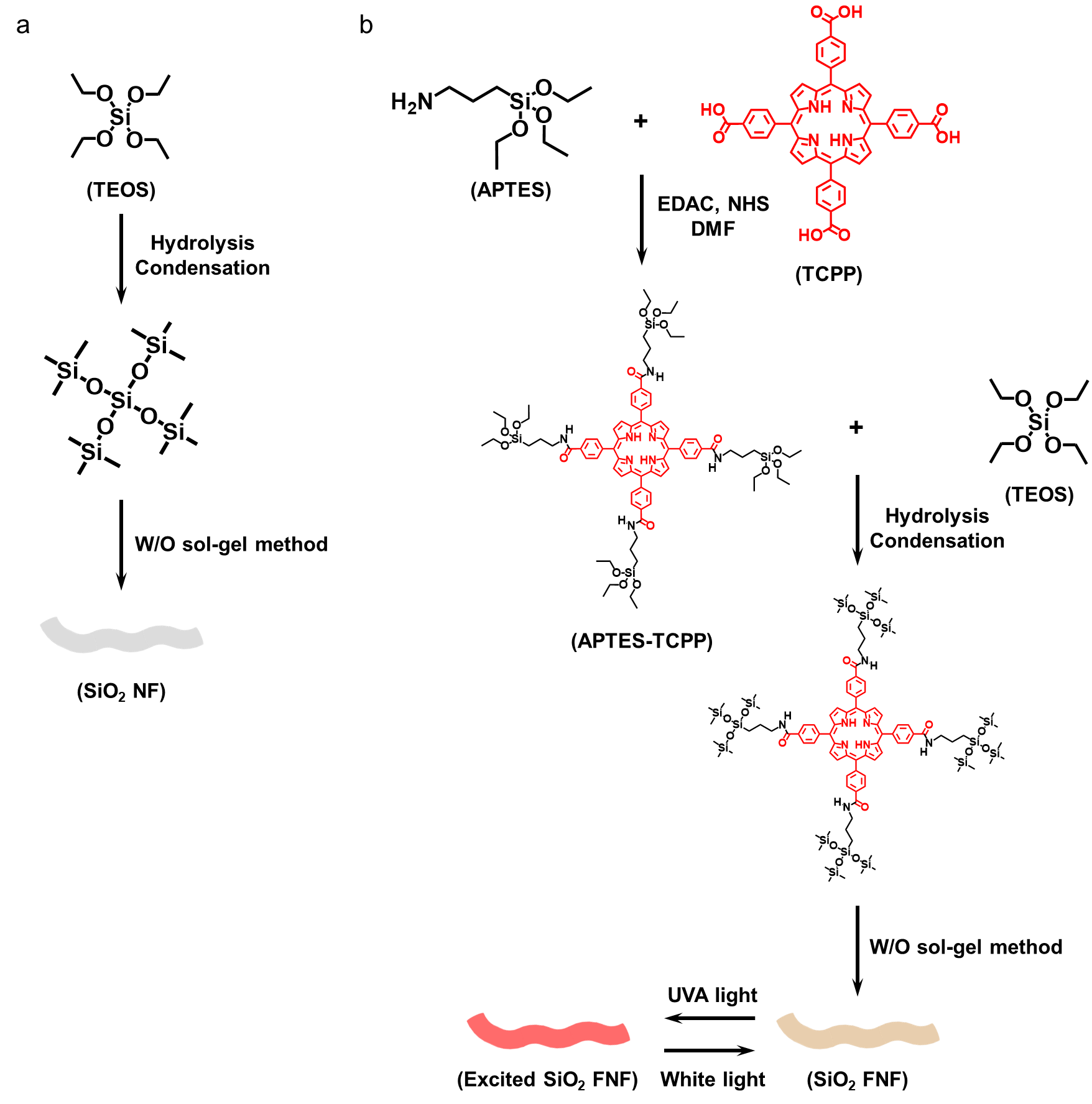


Figure S1. Schematic synthesis routes of (a) silica NFs and (b) silica FNFs.


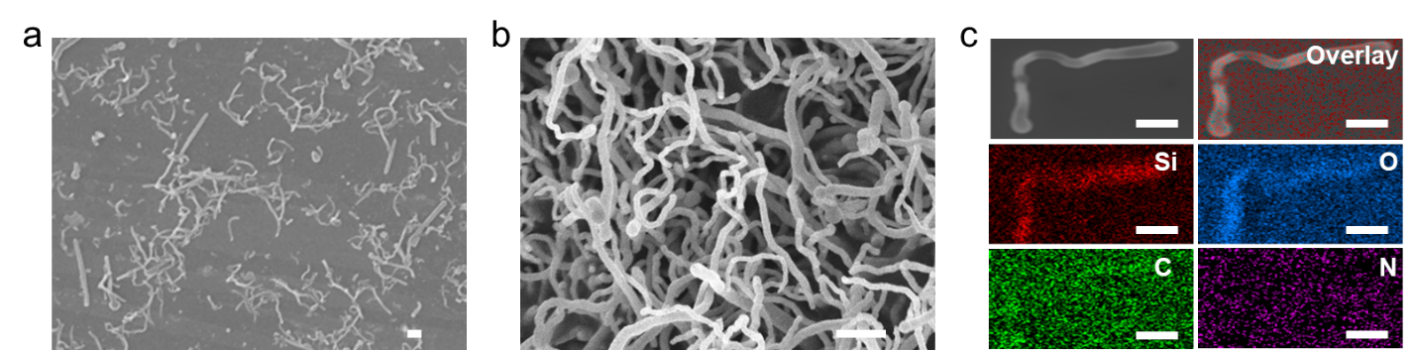


Figure S2. Morphologies of silica NFs. (a) SEM image at (b) a high magnification. The scale bars are 1 μm. (c) Elemental mapping images. The scale bars are 500 nm.


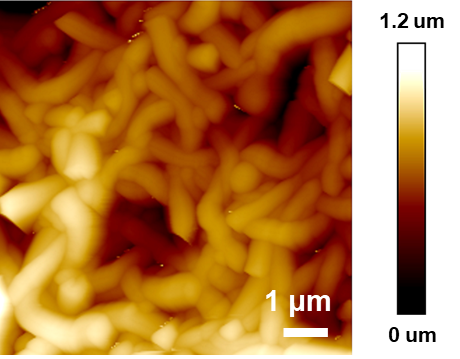


Figure S3. AFM image of silica FNFs.

Figure S4. XRD spectrum of silica FNFs.


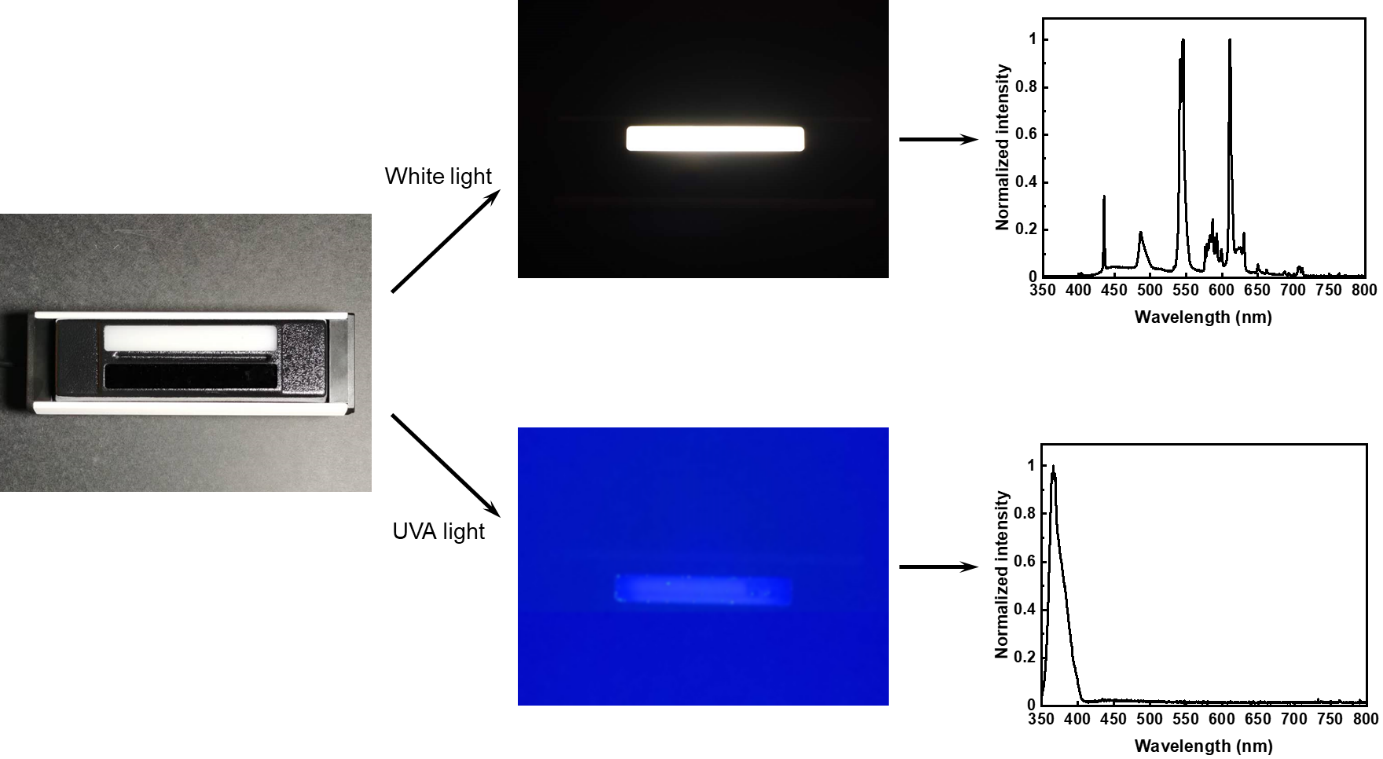


Figure S5. Photographs and spectra of the commercial dual-use 6-Watt illuminator (*Analytikjena UVL-16*) with both mercury-vapor white lamp and UVA lamp.

Figure S6. Relative PL intensity spectra of FNF dispersion under continuous UVA light illumination up to 6 h.


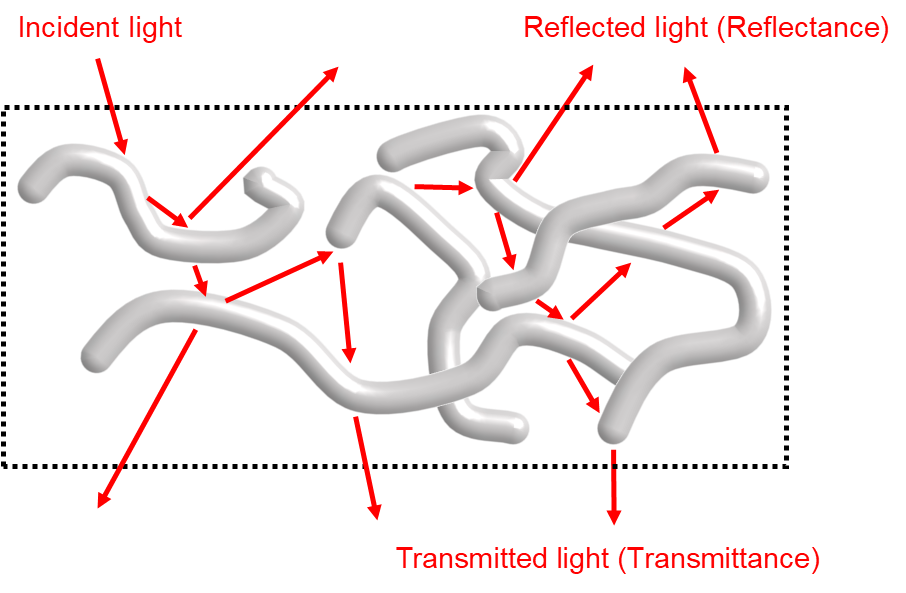


Figure S7. Schematic illustration of light propagation within a porous material composed of NFs.


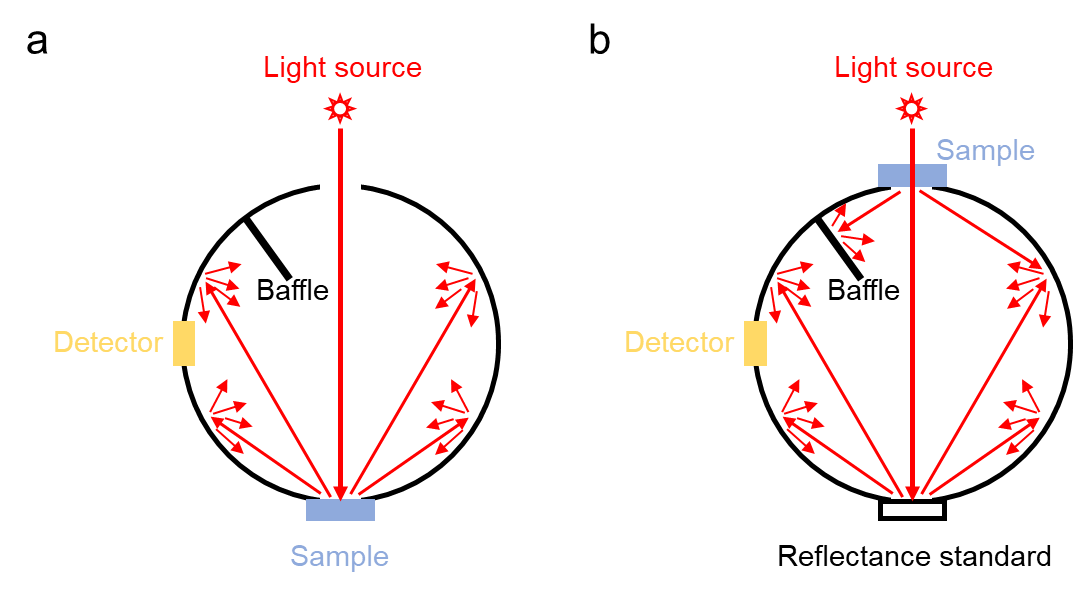


Figure S8. Schematic illustration of (a) reflectance and (b) transmittance tests at different positions of an integrating sphere.

Figure S9. Normalized reflectance of NF film wetted by various organic solvent. “n” is the refractive indexes of the solvents at 20 ℃.


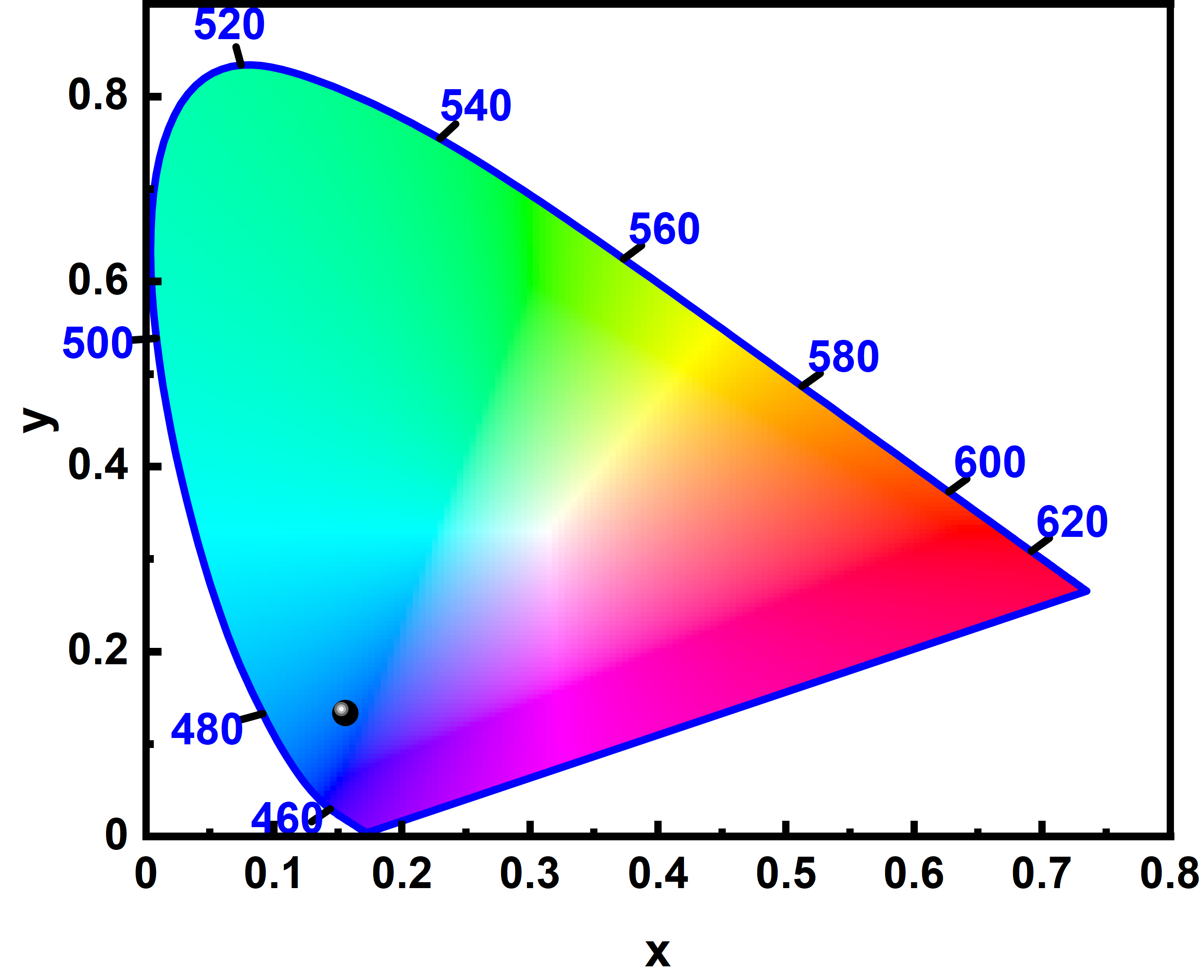


Figure S10. CIE chromaticity coordinate of dry NF/FNF film under UVA light.


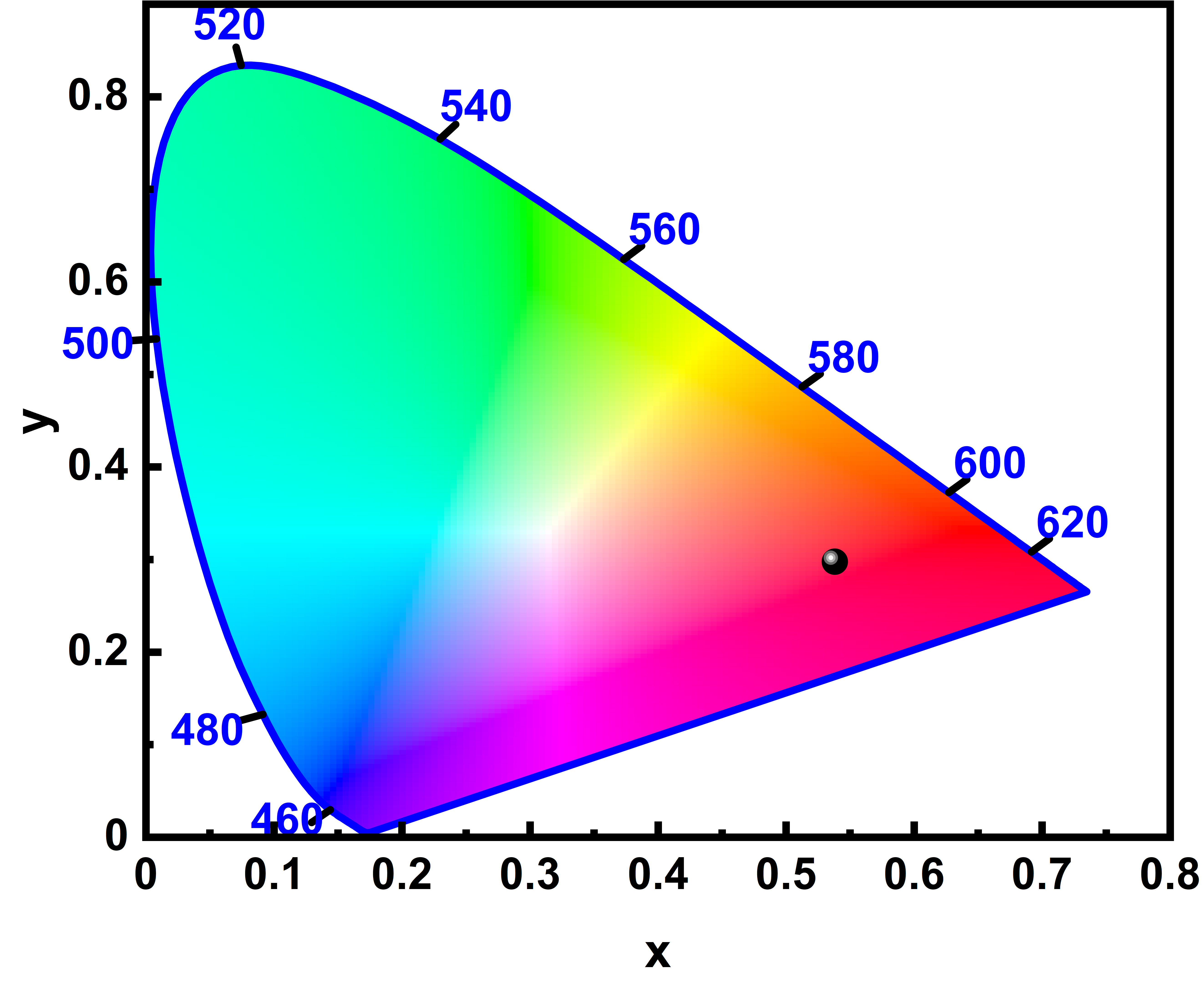


Figure S11. CIE chromaticity coordinate of wet NF/FNF film under UVA light.


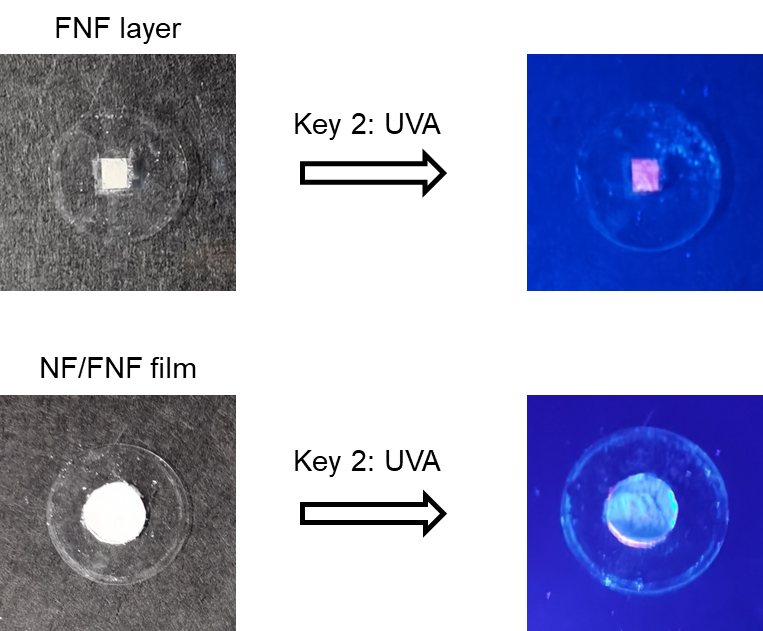


Figure S12. Different encryption security levels without and with silica NF layer shielding.


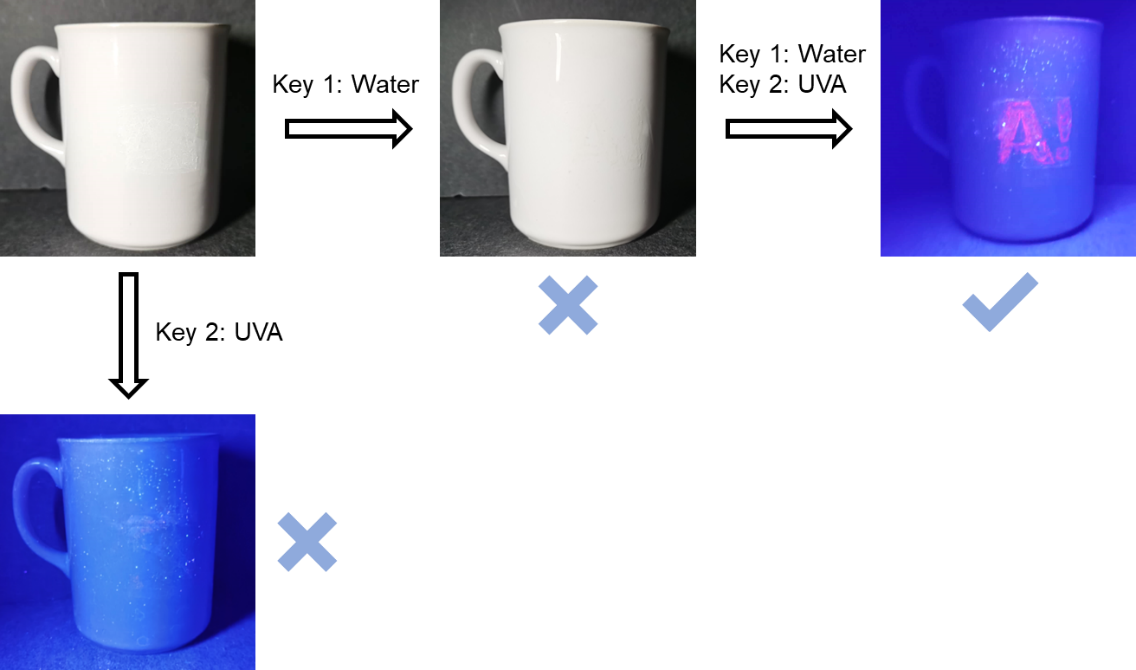


Figure S13. An example of application on the curved surface of a daily-use cup.


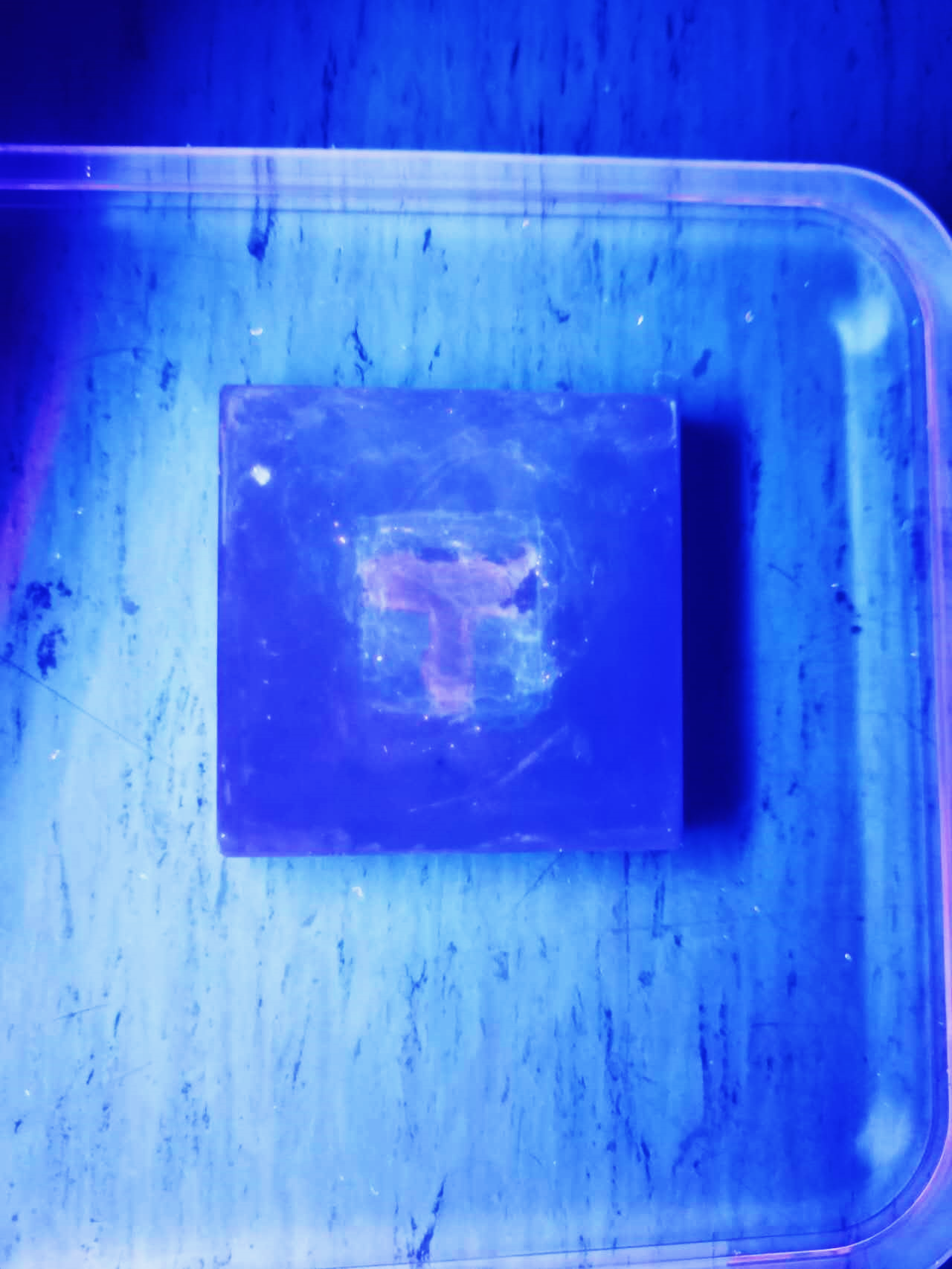


Figure S14. The ceramic sheet (in Figure 6), which has been stored over two years, still reveals the “T” alphabet upon exposure to liquid and UVA light.


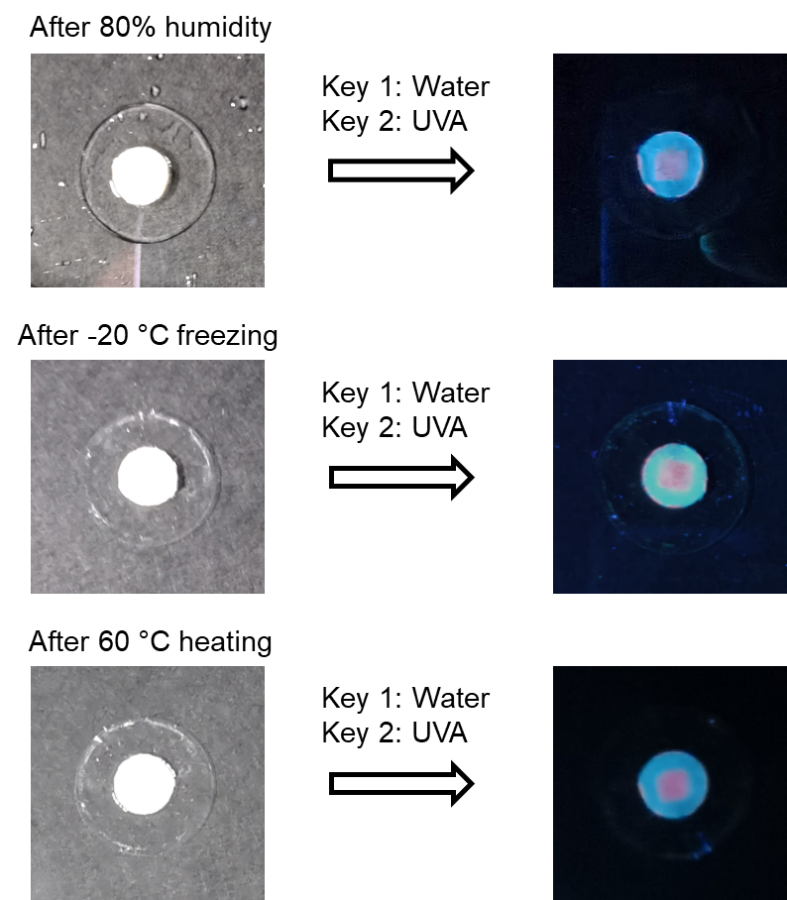


Figure S15. The system remains stable after experiencing different treatments.


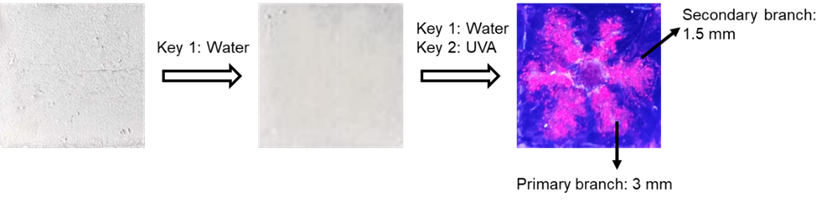


Figure S16. Decryption of a snowflake-shaped pattern featuring hierarchical branched structures. The primary branches (3 mm) are recognizable while the secondary branches (1.5 mm) are blurry.


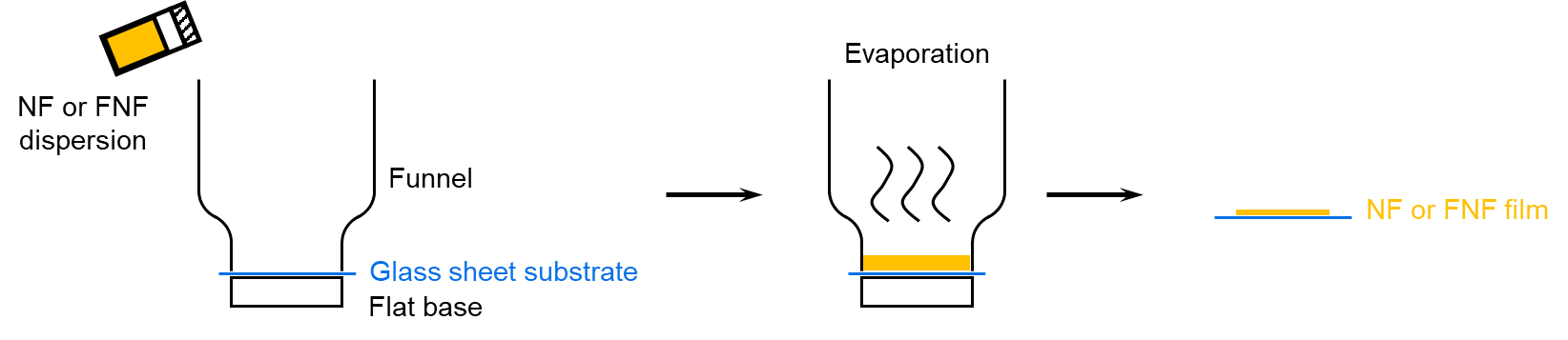


Figure S17. Schematics of preparing NF or FNF film by the evaporation device.

Table S1. Comparison of representative encryption strategies

| Encryption type | Raw material for encryption | System type | Stimuli | Ref. |
| --- | --- | --- | --- | --- |
| Single encryption | Phenol-formaldehyde resin; Cellulose nanocrystal | Photonic film | Liquid | S1 |
| Single encryption | Polydimethylsiloxane; Poly acrylic acid; CdS quantum dot | Inkjet printing | Light | S2 |
| Single encryption | NaYF_4_ nanocrystal; Tm^3+^ | Inkjet printing | Illumination power | S3 |
| Single encryption | Poly(N-isopropylacrylamide); Polyethyleneimine;  Sodium dodecyl sulfate | Hydrogel | Temperature | S4 |
| Double encryption | Polyethylene glycol diacrylate; NaYF_4_ upconversion nanoparticles | Array | Light; Phase-angle | S5 |
| Double encryption | DAS-BA AIEgen | Painting | Light; pH | S6 |
| Double encryption | 2-(4-benzoyl-3-hydroxyphenoxy)ethyl acrylate;  Carbon nanodot; Polyacrylamide; Tb^3+^ | Hydrogel | Light; Force | S7 |
| Multi-color encryption | Poly(hydroxyethyl methacrylate)-Poly(isopropyl acrylamide)-Poly(dimethylamino ethyl methacrylate), Graphene quantum dot; SiO_2_ photonic crystal | Hydrogel | Temperature; pH; Light; Angle | S8 |
| Multi-color encryption | Poly(acrylamide); N,N’-methylenebis(acrylamide); Coumarin; Boron-dipyrromethene; Rhodamine B | Hydrogel | Light | S9 |
| 3D encryption | Gelatin; Poly(acrylamide-acrylate); (E)-N-(4-(2-(2-hydro-  xybenzylidene)-hydrazinecarbonyl) phenyl) methacrylamide; AlCl_3_ | Hydrogel | Fe^3+^; H^+^ | S10 |
| 3D encryption | Poly(vinyl alcohol); Perylenetetracarboxylic acid-grafted gelatin; Fe^3+^; Borax | Hydrogel | H^+^; Light | S11 |
| 3D encryption | Polystyrene; p-Divinylbenzene; Calix[4]pyrrole; Imidazolium; Coumarin; Boron-dipyrromethene; Rhodamine B | Polymer gel | Cl⁻; Light | S12 |
| Dynamic encryption | Poly-(N-isopropylacrylamide-acrylamide);  Poly-(N,N-dimethyl(acrylamidopropyl)ammonium butanesulfonate-acrylamide | Hydrogel | Temperature; Time | S13 |
| Dynamic encryption | 1,6-acylhydrazone decorated pyrene;  1,3-acylhydrazone decorated pyrene | Coating | Solvent; Light; Time | S14 |
| 4D encryption | 2-(2-(9H-carbazol-9-yl) ethyl) isoindoline-1,3-dione;  2-(3-(9H-carbazol-9-yl) propyl) isoindoline-1,3-dione;  2-(4-(9H-carbazol-9-yl) butyl) isoindoline-1,3-dione;  2-(5-(9H-carbazol-9-yl) pentyl) isoindoline-1,3-dione | Painting | Light; Time | S15 |
| 2FA encryption | Silica NFs; TCPP-grafted silica FNFs | Coating | Liquid; Light | This work |

**References**

1. M. K. Khan, A. Bsoul, K. Walus, W. Y. Hamad, M. J. MacLachlan, *Angew. Chem. Int. Ed.* **2015**, 54, 4304−4308.
2. B. Bao, M. Li, Y. Li, J. Jiang, Z. Gu, X. Zhang, L. Jiang, Y. Song, *Small* **2015**, 11, 1649−1654.
3. J. Zhao, D. Jin, E. P. Schartner, Y. Lu, Y. Liu, A. V. Zvyagin, L. Zhang, J. M. Dawes, P. Xi, J. A. Piper, E. M. Goldys, T. M. Monro, *Nat. Nanotechnol.* **2013**, 8, 729−734.
4. L. Bai, Y. Jin, X. Shang. H. Jin, W. Zeng, L. Shi, *Chem. Eng. J.* **2023**, 456, 141082.
5. H. Liu, M. K. G. Jayakumar, K. Huang, Z. Wang, X. Zheng, H. Ågren, Y. Zhang, *Nanoscale* **2017**, 9, 1676−1686.
6. W. Luo, B. Wu, X. Xu, X. Han, J. Hu, G. Wang, *Chem. Eng. J.* **2022**, 431, 133717.
7. Q. Zhu, K. V. Vliet, N. Holten-Andersen, A. Miserez, *Adv. Funct. Mater.* **2019**, 29, 1808191.
8. J. Yan, G. Pan, W. Lin, Z. Tang, J. Zhang, J. Li, W. Li, X. Lin, H. Luo, G. Yi, *Chem. Eng. J.* **2023**, 451, 138922.
9. X. Ji, R. T. Wu, L. Long, X. S. Ke, C. Guo, Y. J. Ghang, V. M. Lynch, F. Huang, J. L. Sessler, *Adv. Mater.* **2018**, 30, 1705480.
10. L. Tang, J. Huang, H. Zhang, T. Yang, Z. Mo, J. Qu, *Eur. Polym. J.* **2020**, 140, 110061.
11. Y. Zhang, X. Le, Y. Jian, W. Lu, J. Zhang, T. Chen, *Adv. Funct. Mater.* **2019**, 29, 1905514.
12. X. Ji, W. Chen, L. Long, F. Huang, J. L. Sessler, *Chem. Sci.* **2018**, 9, 7746−7752.
13. D. Lou, Y. Sun, J. Li, Y. Zheng, Z. Zhou, J. Yang, C. Pan, Z. Zheng, X. Chen, W. Liu, *Angew. Chem. Int. Ed.* **2022**, 61, e202117066.
14. Q. Wang, B. Lin, M. Chen, C. Zhao, H. Tian, D. H. Qu, *Nat. Commun.* **2022**, 13, 4185.
15. X. Wang, H. Ma, M. Gu, C. Lin, N. Gan, Z. Xie, H. Wang, L. Bian, L. Fu, S. Cai, Z. Chi, W. Yao, Z. An, H. Shi, W. Huang, *Chem. Mater.* **2019**, 31, 5584−5591.
